# Successful treatment based on molecular biological assessment of invasive anaplastic lymphoma kinase-positive inflammatory myofibroblastic tumor of the lung

**DOI:** 10.1186/s40792-019-0674-x

**Published:** 2019-07-24

**Authors:** Hironosuke Watanabe, Naoya Yamasaki, Takuro Miyazaki, Keitaro Matsumoto, Tomoshi Tsuchiya, Kuniko Abe, Takeshi Nagayasu

**Affiliations:** 1 Division of Surgical Oncology, Department of Surgery, Nagasaki University Graduate School of Biomedical Sciences, 1-7-1 Sakamoto, Nagasaki, 852-8501 Japan; 2 Department of Pathology, Nagasaki University Graduate School of Biomedical Sciences, Nagasaki, Japan

**Keywords:** Inflammatory myofibroblastic tumor, Anaplastic lymphoma kinase, Immune globulin G 4-related sclerosing disease

## Abstract

**Background:**

Inflammatory myofibroblastic tumor (IMT) of the lung is rare. This disease often shows neoplastic features with anaplastic lymphoma kinase positiveness as well as inflammatory features, such as steroid-responsive immunoglobulin G4 (IgG4)-related sclerosing disease. Since many cases have been reported as advanced, various treatment strategies should be considered based on clinical and biological features of each case.

**Case presentation:**

We report a 49-year-old male with IMT, which seemed invading the left atrium from preoperative imaging modalities. Serological and pathological examinations from the biopsy specimen revealed high expression of anaplastic lymphoma kinase expression in the tumor. On the other hand, IgG4/IgG ration in the tumor was small, where a therapeutic effect of steroid was not expected, leading to surgical treatment rather than a steroid administration. The tumor was completely resected en bloc with the right lower lobe of the lung and a part of the left atrium. The postoperative course of the patient was uneventful. The patient has remained recurrence free over 5 years from the surgery.

**Conclusion:**

In this case, preoperative biological assessment prior to the treatment led to a good clinical course. We believe that molecular biological examination is important in the determination of treatment strategy for this rare disease as well as imaging modalities.

## Background

Inflammatory myofibroblastic tumor (IMT) of the lung is rare and has been called a variety of names, such as inflammatory pseudotumor, plasma cell granuloma, and histiocytoma [1, 2]. It has also demonstrated various clinical features [1], as well as various molecular biological features [2]. Surgical resection has been recommended; however, various treatments including molecular target drug therapy have recently been reported based on molecular and biological features, especially in advanced cases [3].

In this report, we present an asymptomatic but invasive IMT case. We assessed the biological features of the tumor preoperatively and surgical treatment was selected. The postoperative course was uneventful.

## Case presentation

A 49-year-old man was first referred to a local hospital due to a mass on the right hilum found by plain chest computed tomography (CT) during medical check-up. The tumor seemed to be invasive to the left atrium. Therefore, only excisional biopsy by video-assisted thoracoscopic surgery was performed. He was referred to our institution for treatment. His past medical history was unremarkable.

**Fig. 1:**
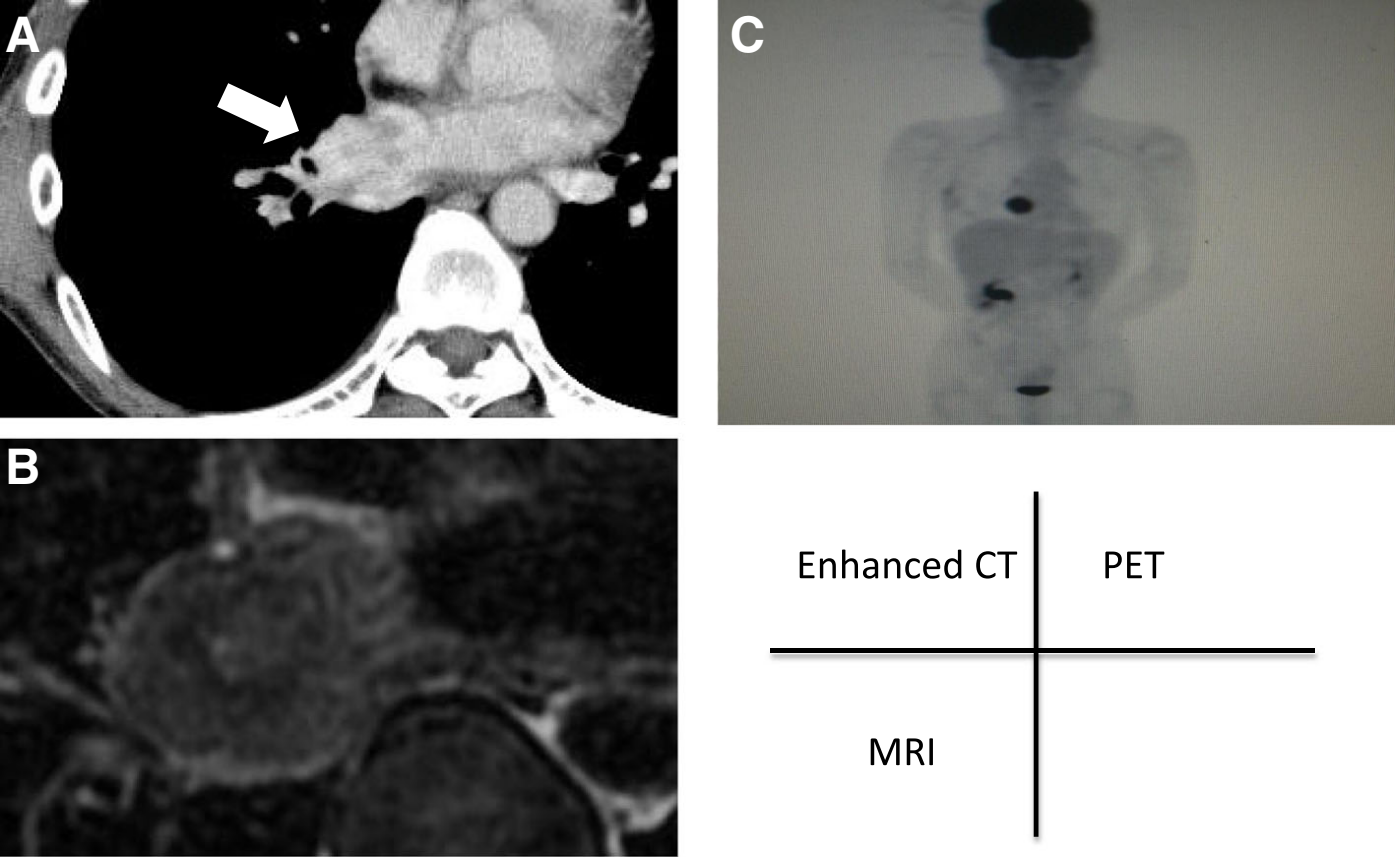
Enhanced chest CT (**a**), MRI (**b**), and PET/CT (**c**). **a** A heterogeneous enhancement was demonstrated within the mass (arrow). **b** Ring-like low signal was shown within the mass. **c** High uptake of fluorodeoxyglucose was demonstrated within the mass

Enhanced chest CT showed a 4-cm mass, with a heterogeneous enhancement (Fig. 1a). Enhanced chest magnetic resonance imaging (MRI) showed a low signal in a T2-weighted image, which indicated the richness of the fibrous components inside the tumor (Fig. 1b). The borderline between the tumor and left atrium was unclear. Positron emission tomography (PET) showed high uptake of fluorodeoxyglucose (FDG) within the tumor lesion (Fig. 1c). All of the tumor markers were within normal limits. We performed the pathological examination with the biopsy specimen from the local hospital, which revealed that there were few cells positive for immunoglobulin G (IgG) 4, one of the subclasses of IgG, of less than 10% (shown in Fig. 1). The serum level of IgG4 was 37.7 mg/dl, and thus the diagnostic criteria of IgG4-related sclerosing disease (IgG4SD), where steroid treatment would be effective, were not fulfilled. On the other hand, anaplastic lymphoma kinase (ALK) expression was positive in the tumor, suggesting the tumor has malignant potential (shown in Fig. 1). We planned to perform an operation because of the possibility of malignancy and a risk of massive bleeding or tumor embolism.

The patient underwent thoracotomy after an isolation of the right femoral artery and vein in preparation for a case of the cardio-pulmonary support that would be needed. The tumor lesion was palpable around the right hilum, surrounding the inferior pulmonary vein (IPV). After resection of the A6, basal artery, and the lower bronchus, the pericardium was opened and the superior and inferior pulmonary veins were isolated in the pericardium. The tumor lesion was revealed to localize at the IPV in the pericardium. Thus, we clumped the left atrium and resected the tumor en bloc with IPV and a part of the left atrium without cardio-pulmonary support. After removal of the tumor, the left atrium was sutured by over and over running suture.

**Fig. 2:**
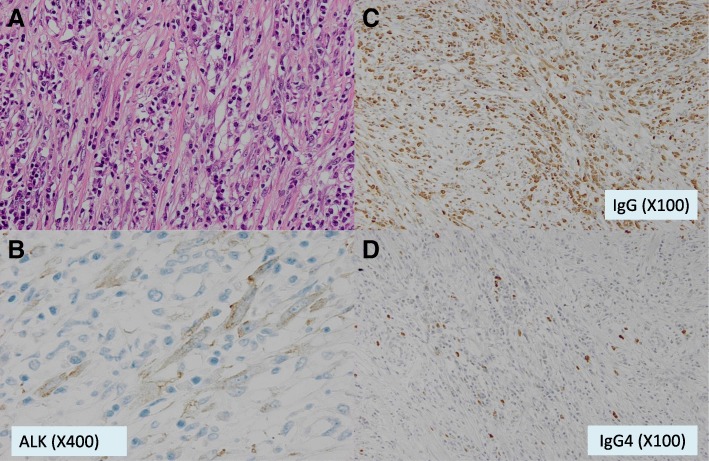
**a** Histopathological examination demonstrated proliferation of spindle cells and collagen fiber, and the infiltration of plasma cells and lymphocytes (× 100). **b** ALK expression was positive in the spindle cells (× 400). **c** There were a lot of IgG-positive plasma cells (× 100). **d** On the other hand, few cells were positive for IgG4-positive plasma cells (× 100)

Pathological examination demonstrated the proliferation of spindle cells and collagen fibers. It also revealed the infiltration of plasma cells and lymphocytes (Fig. 2a). No evidence of malignancy was obtained. Thus, the diagnosis of IMT was gained. Invasion to the IPV, pericardium, and left atrium was observed, but there was no evidence of exposure to the lesion. Smooth muscle actin (not shown) and ALK were positive (Fig. 2b), but the ratio of IgG4/IgG was less than 10% (Fig. 2c, d), the same as that from a previous examination.

The postoperative course of the patient was uneventful. Currently, the patient remains free of disease over 5 years after surgery.

## Discussion

An IMT of the lung is rare, with an incidence of 0.04% [4] to 0.3% [1]. Gender, race, and geographical location appear to play no role in its occurrence [1]. Cerfolio et al. reported that the median age of their case was 47 years (range, 5 to 77 years) [1]. They also reported that the invasive cases were more significantly symptomatic and required more extensive surgery than noninvasive cases. In our case, the patient had no complaint, but the tumor possessed aggressive features. Symptoms may depend on their location, and thus careful analysis on the extensive area of the IMT should be done.

It is unclear whether the etiology of an IMT is the result of non-specific inflammatory reaction or neoplastic change. There were some cases where expression of the ALK gene elevated [5] and others where there was rearrangement of the ALK gene on chromosome 2p23 [6]. In addition, the ALK gene expression was reported to be associated with local recurrence [5]. These reports suggest that an IMT is a neoplastic tumor rather than a result of an inflammatory reaction. On the other hand, IgG4-related sclerosing disease (IgG4SD) has been reported as a steroid-responsive multi-organ disorder with chronic inflammation, and it demonstrates many clinical features, including autoimmune pancreatitis, sclerosing cholangitis, and inflammatory pseudotumor of the lung [7, 8]. The diagnostic criteria of this disease are IgG4/IgG > 0.1 in tissue samples and serum IgG4 concentration > 135 mg/dl [2]. Between IgG4SD and IMT, there are some overlaps including abundance of plasma cells. Recently, IMT demonstrating both ALK high gene expression and highly positive IgG4 cases were reported [2]. This suggests that there may be more etiology and clinical features of IMT.

The 5-year survival rate of IMT was reported 74–91% [1, 4]. Complete resection was recommended [4, 9], and the recurrent cases were related to incomplete resection [1]. Other treatments besides surgery include radiation [1, 10] and chemotherapy [3, 11–13]. Dishop et al. reported a case treated with vincristine and etoposide as the first line and cisplatin, adriamycin, and methotrexate as the second line after incomplete resection [11]. In addition, complete remission was reported using vincristine, ifosfamide, doxorubicin, and celecoxib [12]. However, Trojan reported aggressive IMT case which infiltrated the central nerve system with mediastinal metastases despite chemotherapy including vincristine [13], suggesting the neoplastic feature of IMT. Steroid and non-steroidal anti-inflammatory drugs [3, 11, 12] have also been reported as effective for IMT. Especially, steroids have been reported as effective for IMT containing IgG4SD features [7]. However, steroid was effective even for the case without IgG4SD features [2]. On the other hand, Cerfolio et al. [1] reported two cases where the remaining tumor showed no growth after incomplete resection during 4 to 9 years follow-up and the cases did not receive any additional treatment, although the biological features of those cases were not explained in their article. These findings would make the choice of treatment strategy for IMT complicated. However, since some cases of IMT have shown no malignant feature, observation could be carefully selected based on clinical and molecular biological features of each case. In the present case, because of the possibility of malignancy as well as a risk of massive bleeding or tumor embolism based on the location of the tumor, we performed the surgical resection.

Recently, clinical trial of crizotinib for ALK-positive IMT was reported [3]. In that report, crizotinib administration combined with surgical resection resulted in complete remission in the IMT with ALK rearrangement. However, they also reported that crizotinib was not effective for an ALK-negative case, suggesting that crizotinib might be effective only for ALK-positive IMT cases. In light of the report above, a treatment strategy with or without surgery should be considered carefully based on each feature, especially for invasive cases.

## Conclusion

We reported an asymptomatic but invasive IMT case, which demonstrated the high expression of ALK without IgG4. Because we contradicted the possibility of the IgG4SD beforehand, we chose the operation over other therapies. We believe, to determine the therapeutic strategy and prospect prognosis, analysis of IgG4 and ALK for IMT along with existing imaging modalities may prove useful.

## Data Availability

This article is distributed under the terms of the Creative Commons Attribution 4.0 International License (http://creativecommons.org/licenses/by/4.0/), which permits unrestricted use, distribution, and reproduction in any medium, provided you give appropriate credit to the original author(s) and the source, provide a link to the Creative Commons license, and indicate if changes were made.

## Abbreviations

ALK: Anaplastic lymphoma kinase
CT: Computed tomography
FDG: Fluorodeoxyglucose
Ig: Immunoglobulin
IgG4SD: IgG4-related sclerosing disease
IMT: Inflammatory myofibroblastic tumor
IPV: Inferior pulmonary vein
MRI: Magnetic resonance imaging
PET: Positron emission tomography
